# Is the surgical knot tying technique associated with a risk for unnoticed glove perforation? An experimental study

**DOI:** 10.1186/1754-9493-8-26

**Published:** 2014-06-17

**Authors:** Vincenzo Giordano, Hilton Augusto Koch, Juliano de Sousa Prado, Leonardo Schiavo de Morais, Rafael de Araújo Hara, Felipe Serrão de Souza, Ney Pecegueiro do Amaral

**Affiliations:** 1 Serviço de Ortopedia e Traumatologia Prof. Nova Monteiro, Hospital Municipal Miguel Couto, R. Carlos Góis 375/203 Leblon, 22440-040 Rio de Janeiro, RJ, Brazil; 2 Departamento de Radiologia, Faculdade de Medicina, Universidade Federal do Rio de Janeiro (UFRJ), Rio de Janeiro 21941-590, RJ, Brazil

**Keywords:** Surgical gloves, Sutures, Impermeability testing

## Abstract

**Background:**

The issue of safety in the surgical procedure has recently been widely and openly discussed at the World Health Organization. The use of latex gloves is the current standard of protection during surgery, as they remain intact throughout the procedure. The present study was designed to evaluate the rate of glove perforation during a two-hand technique using polyester sutures in a controlled experimental study.

**Methods:**

Hypothesis was that the gloves used during a two-hand technique using polyester suture suffer punctures. We used 150 pairs of gloves during the experiment. Each investigator performed 30 tests always using double gloving. They made five surgical knots on each test over a custom-made table specifically developed for the experiment. Ten tests were done at a time with a week- interval. The Control Group (CG) has 30 pairs of intact surgical gloves. The gloves were tested to impermeability by water filling and leaking was observed at three different times. Statistics relating to the perforation rate were analyzed using the chi-square test. A P value less than 0.05 was considered statistically significant.

**Results:**

During the experiment there was no loss of gloves by drilling or inadvertent error in performing the impermeability test. No perforations were detected at any time during the impermeability test with the gloves used for sutures. Also, the CG presented no leakage of the liquid used for the test. There was no statistical difference between the groups underwent suture nor between them and the GC.

**Conclusion:**

Under the studied conditions, the authors’ hypotheses could not be proved. There was no damage to the surgical gloves during the entire experiment. The authors believe that the skin abrasions observed in the ulnar side of the little finger, constant throughout the experiment, must be caused by friction. We feel there is no risk of perforation of surgical gloves during a two-hand technique using polyester suture.

## Introduction

The issue of safety in the surgical procedure has recently been widely and openly discussed at the World Health Organization (WHO) [1]. Despite years of experience with the called “modern surgery”, yet are observed complications and avoidable errors during the pre and intraoperative phases. With that, come many emerging protocols to protect the patient, the surgical team and staff working within the surgical center [1].

In this context, the contamination during the operative procedure has highlighted importance, since its occurrence potentially put at risk of infection to both the patient and staff that performs surgery. Some relatively simple procedures such as adequate preoperative evaluation in order to identify a pre-existing diseases and proper hand hygiene during asepsis moments before the procedure, are fundamental and can avoid more serious problems that may put lives at risk [1].

During surgery, using sterile gloves acts as a protective barrier against pathogens present in blood and skin, such as human immunodeficiency virus and hepatitis B and C and several common bacteria [2-9]. Because of its importance and because of the risk of perforation during surgery, the use of two gloves has generally been adopted by most surgical teams in numerous situations as a way to reduce the risk of perforation, although this measure was not included in WHO recommendations on safety in surgery, since there are no sufficient studies to prove their efficacy [3,4,7,8].

The use of latex gloves is the current standard of protection during surgery, providing enough security to the patient and surgical team, as they remain intact throughout the procedure [2-9]. However, several conditions have been linked to increased risk of glove perforation during surgery, such as frequent handling of sharp instruments and prolonged procedures. In the literature, the highest rates of glove perforation are observed in orthopedic and trauma-thoracic procedures, occurring in up to 61% of patients [3].

Over the years, the authors have observed that the use of polyester sutures using both hands causes intraoperative discomfort and the appearance of small cracks in the fingers, especially the small finger, almost immediately after the surgical procedure. The presence of skin lesions put on alert about the possibility of having a violation of the protective barrier between the patient and surgical team members. However, to our knowledge, there is no evidence whether or not glove perforation caused by friction of the polyester suture during the procedure with this technique.

The authors’ hypothesis is that the gloves used during a two-hand technique using polyester suture suffer punctures. The present study was designed to evaluate the occurrence of glove perforation in an experimental model controlled.

## Methods

### Development of experimental model

**Figure 1:**
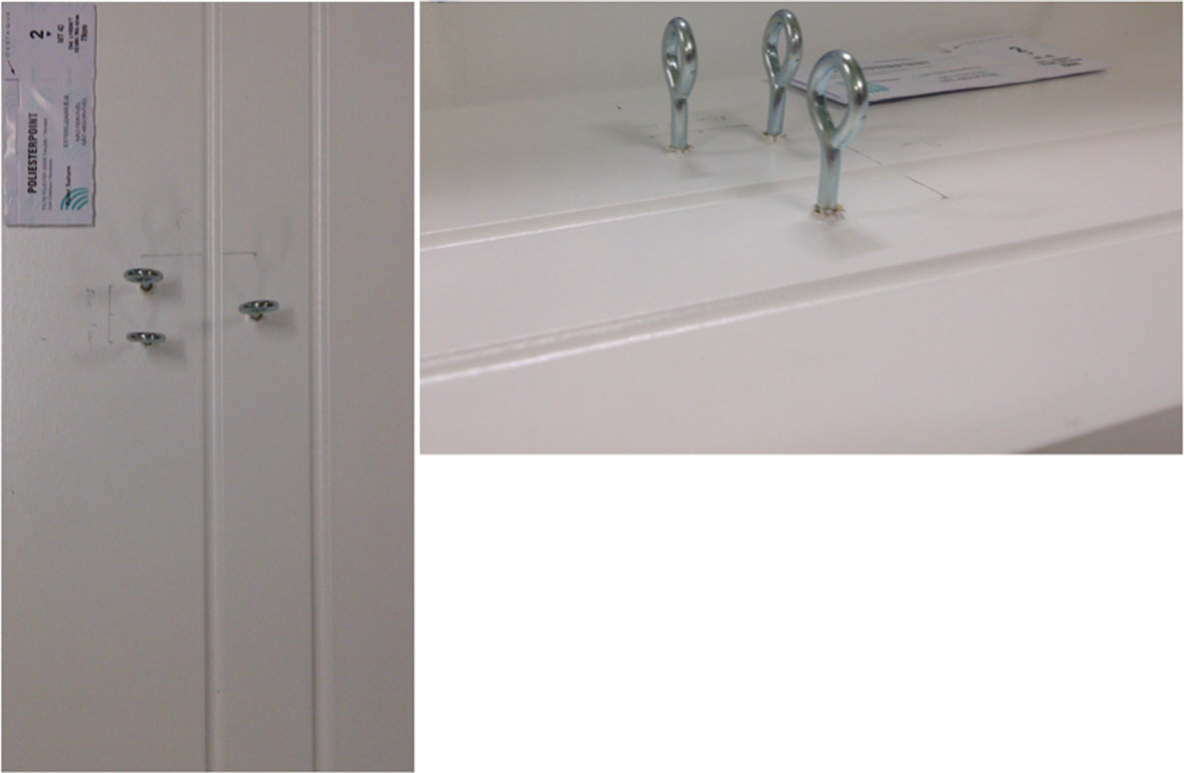
**Bench test used to perform the experiment.** Note in the right picture the arrangement of the rings (triangular) to allow performing the sutures at the base.

**Figure 2:**
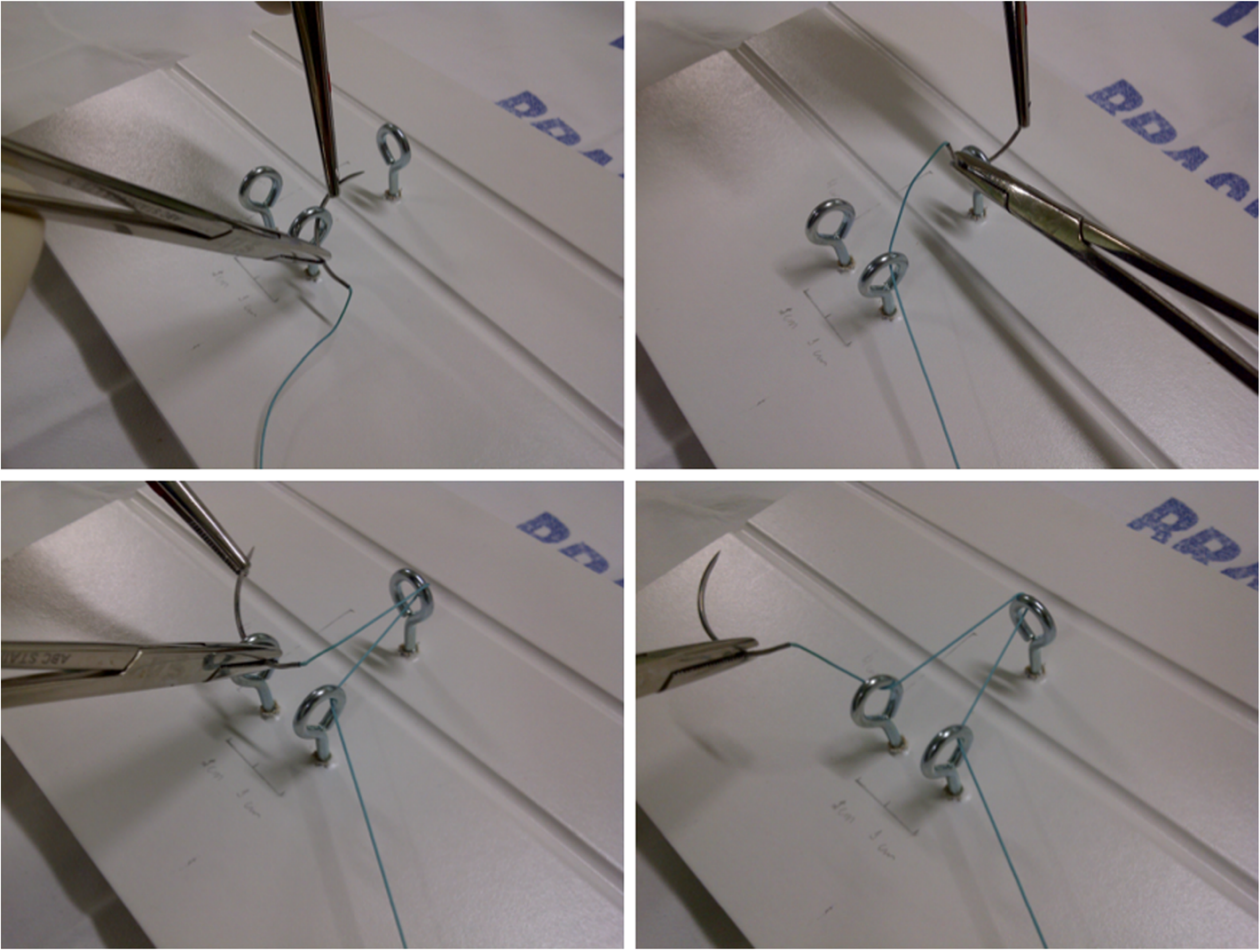
Sequence of suturing using the triangular base as the place of surgical knots.

At the laboratory, experimental model was designed using a suture test bench, which has been done one centimeter defect to be sutured (Figure 1). To the suture support, was used fixed rings arranged in a triangular shape to allow the completion of surgical knots always based on previous configuration. Polyester sutures were used and two brands of latex surgical gloves. The polyester suture was 2/0 Pariéster (Paramed Medical Devices LTDA, São Paulo, Brazil) and gloves of two brands: size 8.0 Lemgruber (Frontinense Latex Industry S/A, Rio de Janeiro, Brazil) and size 8.5 Sanro (Factory Artefatos Latex São Roque, São Paulo, Brazil). A needle holder and a clamp-tooth rat were using for the passage of the wire through the rings until the moment of the surgical knots (Figure 2).

### Sutures

**Figure 3:**
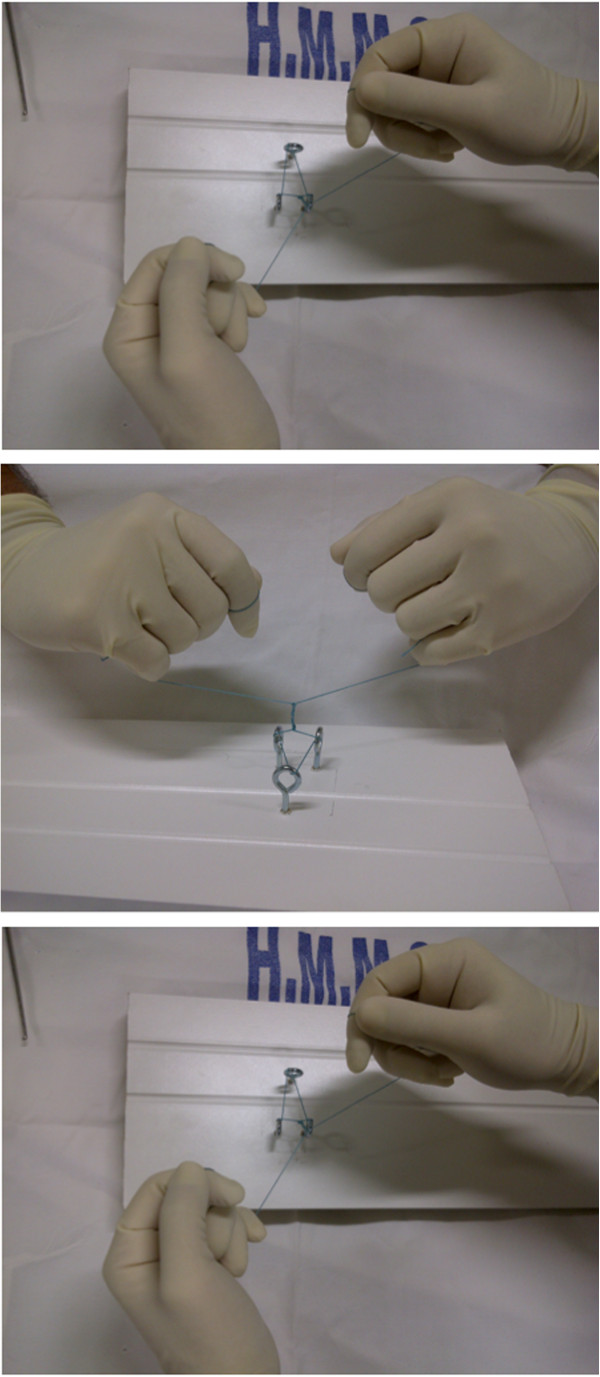
**Performing the surgical suture with five surgical knots each time.** The technique used was the double hand (without needle holder).

**Figure 4:**
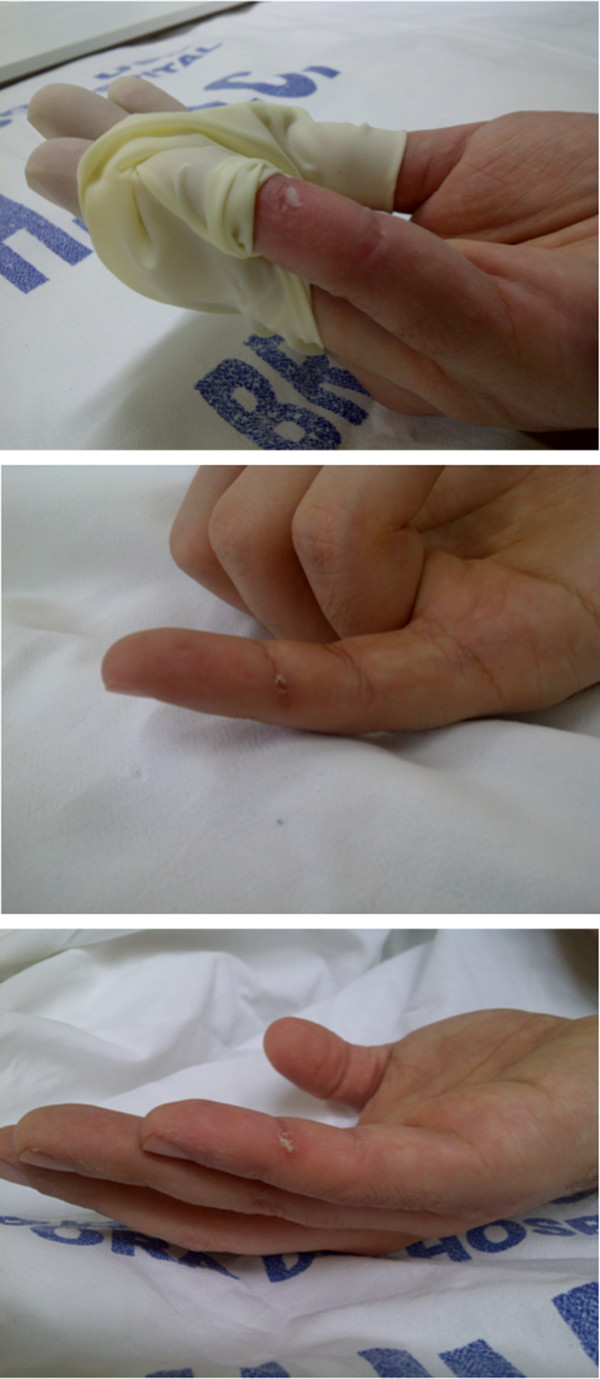
**Injury occurred on the little finger of investigators’ hands during the experiment.** Note the highest to lowest image the evolution of the lesion from the first to the third week. In the third week clearly notes a skin wound healing in the injured finger.

Sutures forces always performed by two investigators, using two sterile gloves. Were performed five surgical knots in each test (Figure 3) [10]. Due to the occurrence of injury on the fingers during suturing by hand, the tests were divided into three phases to researchers heal their fingers and do not reduce the strength of the sutures (Figure 4). The interval between one phase and another was one week. At each phase, researchers performed 10 tests each. At the end of each experiment, the gloves were identified as the side (right and left) and hand position (inner and outer), placed in plastic bags for protection and stored until the time of the impermeability test.

### Impermeability testing

The impermeability testing followed the Board Resolution RDC in 05/2008 ANVISA (National Health Surveillance Agency) – Evaluation rules for conformity of surgical gloves in Brazil – Normative Reference: ISO 10282/2005 – Surgical Glove [11].

The gloves were filled with a colored solution composed of 450 ml of water and 3 g of aniline dye to facilitate identification of slits and perforations. The volume filled the gloves to the height of the wrist. Next, gloves were closed with a knot and placed upright in a holding device 24 hours. Were observed three distinct leak moments: the first (1^st^ time - T1) immediately after the placement of gloves in a vertical position, the second (2^nd^ time - T2), two to four minutes, and the third and last time (3^rd^ time - T3), after 24 hours, when the gloves were removed from the holding device.

We used other 30 pairs of gloves, 15 of each brand, as a control group (CG). The permeability test by water addition was made in these gloves at the same time.

### Statistical analysis

The existence of slits and perforations was analyzed statistically using the chi-square test considered significant when P less than 0.05 [12].

## Results

During the experiment there was no loss of gloves by drilling or inadvertent error in performing the impermeability test.

A total of 120 inner and outer gloves were used for the suturing test and 60 unused gloves were used as controls. The gloves used for the tests were collected in bins marked right and left hand and inner and outer glove. No perforations were detected at any time during the impermeability test with the gloves used for sutures. It was used aniline dye in order to facilitate the identification of any puncture. There was no extravasation of the blue dye both in the inner and the outer glove demonstrating no perforation area.

Sixty unused gloves were used as controls. In the control group there was no leakage of the liquid used for the test, showing no pre-existing glove perforation. This finding was similar to the recommended by the Brazilian standard specification for sterile gloves, which is one out of 500,000 [13].

There was no statistical difference between the groups underwent suture nor between them and the GC.

Skin abrasions were detected in both surgeons’ hands. It was always found in the ulnar side of the little finger. The presence of skin damage was attributed to local friction against the branded suture.

## Discussion

For years exposure to blood, other body fluids and different microorganisms that compose the natural microflora of humans is recognized as a potential cause of contamination between the health professional and the patient. The use of gloves during procedures where there is exposure to these substances reduces the risk of contamination and spread of diseases between patient and medical staff. Historically, its use dates back to the late 19^th^ century, initially for medical protection when dealing with infectious diseases and later extending to the patient who needed invasive intervention [6].

Currently, although its use does not find defined support in the literature, the use of two surgical gloves on each hand has been widely accepted as a way of increasing the protective barrier and reduces the chance of direct contact between the patient and the doctor [3,4,7,8]. Despite this apparent protection increasing, certain intraoperative situations continue to decrease the integrity of the gloves. The needle perforation is the most frequent cause of contamination during surgery [6,9]. Specifically in orthopedics, the presence of bone fragments and osteosynthesis material increases the risk of glove perforation [3]. The incidence of damage to the surgical gloves varies according to the orthopedic procedure performed, in pediatric surgery was lower (about 14%) and higher in osteosynthesis and hip surgery (above 50%) [9]. The existence of previous injury on the fingers or hands raises the risk of contamination, in case occurs a penetration of infected material during invasive procedures [8].

Epidemiological data indicates that the average probability of a disease transmission after perforation by needles or other sharp instruments varies from 0.2% to 0.5% for the human immunodeficiency virus in adults, from 30% to hepatitis B virus and from 5% to 10% for the hepatitis C virus [5]. It is believed that breaking the protective barrier imposed by surgical glove, place the surgeon or another member of his team at risk for more than one infection by hepatitis during his life. As for the human immunodeficiency virus in adults, it is estimated that one in 1,500 surgeons will be infected in the next 15 years due to glove perforations [14].

This fact motivated the authors to carry out this experiment, given the existence of cuts and abrasions on the fingers during and immediately after the use of polyester sutures in surgical procedures. The hypothesis was that the sutures would be able to cut the fingers in the regions of maximum support and where perform most of the force during the procedure. However, the test of impermeability by adding water, ANVISA standard for tests of INMETRO (National Institute of Metrology), revealed that the gloves did not suffer any damage nor perforations during the performance of surgical knots using our idealized technique model [11]. Consequently, we can assume that the injury occurred on the fingers was caused indirectly by friction and subsequent abrasion of the skin.

Potentially questionable points of our experiment that can be tested in future investigations are using another type of braided suture or the polyester of a larger diameter (5/0) and not performing the impermeability test by blowing air. We believe that using a greater diameter sutures and including a second test may achieve more comprehension on the observed study results.

## Conclusion

Under the studied conditions, the authors’ hypotheses could not be proved. There was no damage to the surgical gloves during the entire experiment. The authors believe that the skin abrasions observed in the ulnar side of the little finger, constant throughout the experiment, must be caused by friction. We feel there is no risk of perforation of surgical gloves during a two-hand technique using polyester suture.

## Competing interests

There are no known conflicts of interest associated with this publication and there has been no significant financial support for this work that could have influenced its outcome.

## Authors’ contributions

VG and NPA designed the study and evaluated the results and drafted the first version of the manuscript. VG, JSP, LSM, and FSS performed the experiments. HAK contributed to revisions of the manuscript. All authors read and approved the final version of the manuscript.
